# Ocular complications with the use of radium-223: a case series

**DOI:** 10.1186/s13014-022-02060-z

**Published:** 2022-05-17

**Authors:** Julie R. Bloom, Alexandra G. Castillejos, Brianna Jones, Nimesh Patel, Barry S. Rosenstein, Richard G. Stock

**Affiliations:** 1 Department of Radiation Oncology, Icahn School of Medicine, Mount Sinai Hospital, 1184 5th Ave, 1st Floor, New York, NY 10029 USA; 2 Department of Ophthalmology, Harvard Medical School, Massachusetts Eye and Ear Infirmary, 243 Charles St, Boston, MA 02114 USA

**Keywords:** Ocular toxicity, Ophthalmology, Radium-223, Radiation Therapy, Uveitis, Hyphema, Prostate cancer, Adverse events, Polypharmacy

## Abstract

**Background:**

Radium-223 is used for the treatment of osseous metastases in castrate-resistant prostate cancer, and has been shown to increase time to the first skeletal-related event, reduce the rate of hospitalization, and improve quality of life. It is well tolerated, with hematologic toxicity as the main adverse event. Thus far, no ocular complication has been reported in the literature after initial administration of radium-223 with a single case reported of ocular complications after a patient’s second course of radium-223.

**Case presentations:**

We present three cases of ocular complications after the use of radium-223 in patients with metastatic prostatic adenocarcinoma. Ocular complications presented as blurry vision, and formal diagnosis included uveitis and hyphema.

**Conclusions:**

Documentation of adverse events is exceedingly important due to the high incidence of metastatic prostate cancer and increasing interest for the use of radium-223 in other osteoblastic disease. The authors postulate that these ocular complications may be a result of radiation’s potential effect on neovascularization, polypharmacy, or the biomolecular effects of radium-223 on integral signaling proteins, potentially coupled with poor underlying ocular health.

## Background

Radium-223 dichloride (Ra-223) is an alpha-emitting radionuclide, approved for treatment of osseous metastases in castrate-resistant prostate cancer, without known visceral metastases. The use of Ra-223 has been shown to improve overall survival, bone pain, increase time to the first skeletal-related event, reduce rate of spinal cord compression, hospitalization, improve quality of life, and decrease the use of external beam radiation therapy [1–5].

As an alkaline earth element, similar to calcium, Ra-223 accumulates in areas of increased bone remodeling [6]. At sites of active bone formation, Ra-223 is used in place of calcium as part of the calcium hydroxyapatite matrix [7]. It has a half-life of 11.4 days; with each decay, it emits four alpha-particles with high linear energy transfer of 27.4 MeV [8]. The short range (~ 100 micro meters (um)), or maximum distance traveled, of alpha particles allows local deposition of energy in bone producing cytotoxic, double-strand DNA breaks in tumor cells, while minimizing adverse normal tissue effects [9].

Ra-223 is extremely well tolerated and poses acceptable toxicities. In a randomized trial of Ra-223 vs placebo, Ra-223 presented with fewer adverse events (AEs), less grade 3 or 4 toxicity-related AEs, and less serious AEs [1]. The most common side effects are hematologic including anemia, thrombocytopenia, and neutropenia, secondary to the impact of radiation on the bone marrow [1]. The most common non-hematologic side effects include bone pain flare, fatigue, nausea, and diarrhea [10]. To date there has been few documented ocular toxicities in humans related to Ra-223 [11]. In the early assessment of Ra-223, single injections of high-dose Ra-223 in canines resulted in retinal detachment, thought to be due to increased uptake of alpha radiation in pigmented epithelium due to its increased number of calcium binding sites. However, humans do not have this epithelial layer, the tapetum lucidum. No cases of retinal detachment or optic toxicities have been reported in clinical trials [12].

Ocular complications from local radiation therapy are varied, and dependent on specific parameters including total radiation dose, dose volume, dose rate, proximity to macular and optic disc, and biologic variable response [13]. Acute toxicities include uveitis, conjunctivitis, iridocyclitis, keratitis, and transient myopia. Late toxicities include dry eye, cataract formation, corneal ulceration, nasolacrimal duct obstruction, neovascularization of the iris or the optic nerve head, neovascular glaucoma, optic neuropathy, and retinopathy [14, 15]. These reported ocular complications may occur when the eye is directly in the field of radiation therapy, treated with external beam radiation therapy or brachytherapy. For patients who receive plaque brachytherapy, dry eye may be experienced in up to 9% of patients, keratitis in 4–21% of patients, and iris neovascularization and neovascular glaucoma in 4–23% of patients. [16, 17] Radiation-induced retinopathy ranges from 10 to 63% [18]. Optic neuropathy which may present as sudden, painless monocular vision loss may occur between 8 to 46% of patients at 5 years, dependent on tumor size [19].

Here we present three cases in which Ra-223 may have had a detrimental effect on ocular health, see Table 1.

**Table 1.** Case characteristics, timeline and outcome

Patient	Characteristics	Timing of ocular symptoms	Presentation	Formal diagnosis	Treatment	Resolution
1	74 M, G9 (4 + 5), ATM and BRAF mutations, cardiac comorbidities, bilateral cataracts and bilateral ocular hypertension, prior Ra-223 treatment	2 weeks after first cycle of Ra-223	Bilateral blurry vision	Anterior uveitis, hyphema of the left eye	Prednisolone acetate 1% 4× daily, atropine 1% daily and brimonidine 0.2% daily	Bilateral anterior uveitis and left eye hyphema resolved within 2 months
2	78 M, G9 (4 + 5), history of right eye strabismic amblyopia, bilateral macular drusen, bilateral narrow angles; no genetic pathologic mutations	1 week after first cycle of Ra-223	Monocular blurry vision	Posterior capsular opacification (PCO) with stable drusen	Laser capsulotomy (underlying PCO unlikely related to Ra-223)	Partial improvement in blurry vision
3	74 M, G7 (4 + 3), history of mild cognitive impairment; concurrent use of enzalutamide	48 hours after first cycle of Ra-223	Bilateral blurry vision	Normal eye exam	None	Blurry vision stabilized after 2 weeks

## Case presentations

### Case 1

Patient 1 is a 74 year-old male with a history of coronary artery disease, inferior myocardial infarction status-post stent placements, aortic valve stenosis, bilateral cataracts, bilateral ocular hypertension, and prostate cancer (PCa), pT3b, Gleason 9 (4 + 5) with pre-treatment prostate-specific antigen (PSA) 14.2 ng/mL. He underwent robot-assisted laparoscopic prostatectomy (RALP) in June 2013 with subsequent biochemical failure, and received salvage external beam radiation therapy (EBRT) to 70.2 Gy in 39 fractions with adjuvant androgen deprivation therapy (ADT). He developed mCRPC to the bones and initiated abiraterone 3 years later; he subsequently switched to enzalutamide. Two years later, he was started on apalutamide, and completed Ra-223, 6 cycles. He received further systemic therapy including docetaxel and olaparib, after being found to have ATM and BRAF mutations.

The patient then started a second course of Ra-223; two weeks after the patient’s first cycle, he presented to the emergency room with new onset bilateral blurry vision. The patient was found to have anterior uveitis and hyphema in the left eye for which he was started on prednisolone acetate 1% four times daily, brimonidine 0.2% and atropine 1% once daily in the left eye. During a later outpatient ophthalmology visit, visual acuity in the right eye (OD) was noted to be 20/30 and in the left eye (OS) 20/60, while intraocular pressure was 23 and 13, respectively. Slit lamp examination revealed trace white cell and cataract in the right eye; left eye had a quiet anterior chamber with few iris synechiae, evidence of resolved left eye anterior uveitis, as well as a cataract. Gonioscopy revealed the patient was open to the ciliary body in both eyes with no evidence of neovascularization of the iris or angle, with inferior layering hyphema found only in the left eye. Fundus examination showed a healthy cup to disc ratio of 0.3 in both eyes and bilateral epiretinal membranes (ERM). The patient was continued on the previous drops and sent for basic uveitis lab workup; however, the patient never underwent this testing due to systemic issues and hospitalizations.

With continuation of the above treatment his bilateral anterior uveitis and left eye hyphema resolved within 2 months; however, he continued to have spikes in intraocular pressures in both eyes for which brimonidine was switched to latanoprost 0.05% nightly in both eyes with improvement in intraocular pressures. The patient's visual acuity at last follow-up was 20/40 in both eyes.

### Case 2

Patient 2 is a 78 year-old male with a history of hypertension (HTN), hyperlipidemia, right eye strabismic amblyopia, bilateral macular drusen, bilateral narrow angles status post laser iridotomy, and PCa initially diagnosed. He received definitive EBRT, but was unfortunately diagnosed with biochemical failure and maintained on bicalutamide for 10 years. Twelve years after initial diagnosis, he was found to have radiographic evidence of osseous metastatic disease and received treatment including ADT, EBRT to sites of osseous disease, enzalutamide, abiraterone, and darolutamide.

While maintained on ADT and denosumab, he initiated Ra-223. One week after the infusion, he experienced transient left eye blurry vision. At that time, he was taking tadalafil 5 mg daily, amlodipine 10 mg daily, ibuprofen 600 mg daily, omeprazole 40 mg daily, mirabegron 50 mg daily, darolutamide 300 mg daily and alfuzosin 10 mg daily. There were no recent changes in medications or dosing. After the 5th infusion, the patient was seen by his ophthalmologist, his macular degeneration was noted to be stable. He had a best corrected visual acuity of hand motion OD and 20/40 OS. Intraocular pressure was 13 OD and 15 OS. Slit lamp exam revealed bilateral laser peripheral iridotomies as well as a 1 + posterior capsular opacity OS for which he underwent yttrium–aluminum-garnet laser capsulotomy. Per the patient, he experienced a moderate improvement in his vision, and after capsulotomy visual acuity had improved to 20/25 OS. Bone scan after his 6^th^ Ra-223 infusion demonstrated stable osteoblastic lesions.

### Case 3

Patient 3 is a 74 year-old male with a history of mild cognitive impairment, diabetes mellitus type II (HbA1c 8.7, insulin-dependent), HTN, and Stage IIIB, pT3bN0 Gleason 7 (4 + 3) PCa. He underwent RALP and lymphadenectomy, and, ultimately, salvage EBRT with concurrent ADT. Three years later, he was diagnosed with osseous metastatic disease and was started on intermittent ADT. He was ultimately enrolled in a clinical trial and initiated study pharmaceuticals including abiraterone, cabazitaxel, and carboplatin. He was scheduled for Ra-223 as part of the trial with concurrent ADT.

Forty-eight hours after undergoing the first infusion of Ra-223 with concurrent enzalutamide 40 mg oral tablet, he noted new mild, bilateral blurry vision. The patient was referred to an ophthalmologist where ocular exam revealed a visual acuity of 20/25 OD, no visual acuity was notated for the left eye. Intraocular pressures were normal, 15 bilaterally. Anterior exam showed white and quiet conjunctiva, clear corneas, and deep and quiet anterior chambers, while fundus exam was found to be unremarkable. Auto-perimetry visual field testing was normal in both eyes. The patient was prescribed a new pair of glasses for his myopia with no further ophthalmology follow up to date. Within weeks, the blurry vision stabilized. Follow-up bone scan one month later revealed stable multiple osseous metastatic disease.

## Discussion

These cases present an interesting association between patients’ receiving Ra-223 for mCRPC and ocular changes. All three cases presented with blurry vision as the main ocular symptom with blurry vision resolving or stabilizing within 2 months in all patients. All ocular complications occurred after the first cycle, while timing of onset varied between 48 hours and 2 weeks after the first cycle.

To our knowledge, only one previous study has demonstrated ocular sequalae in patients who received Ra-223, although this was after *retreatment* with Ra-223 [11]. In this phase I/II study assessing retreatment with Ra-223 for mCRPC, ocular AEs including cataract formation, cataract worsening, iritis, uveitis, glaucoma, and photopsia occurred in 11% (5/44) of patients. Two patients had a history of uveitis and glaucoma, in addition to diabetic retinopathy and other risk factors. All ocular events were considered unrelated to Ra-223 by the investigator except for one case of grade 1 photopsia.

### Uveitis and hyphema with the use of radiation

The first case presented here shows an example of bilateral uveitis and unilateral hyphema in a patient with no known prior history of either condition. Uveitis can be seen as an acute dose-dependent effect of radiation therapy thought to be caused by an increase in vascular permeability leading to permeation of inflammatory proteins and cells [14]. However, the etiology of this patient’s hyphema remains unclear.

Hyphema—the collection of blood in the anterior chamber of the eye—is most commonly secondary to blunt or penetrating trauma with additional causes including local neoplasm, uveitis, juvenile xanthogranuloma, coagulopathies, post-surgical, neovascularization of the iris, or anterior segment ischemia [14]. Neovascularization of the iris, in the context of local radiation therapy, is dose dependent and usually occurs on the time frame of months to years after the initiation of therapy [20]. Hyphema formation has been reported in patients treated with both local plaque brachytherapy and ocular proton beam radiation therapy, but has not been reported with use of systemic, particle radiation therapy [21–23].

In this first case, the hyphema occurred shortly after radiation therapy and no neovascularization of the iris or angle was noted on exam. A limited view during the acute phase of a hyphema could obscure evidence of neovascularization at the time of examination leaving neovascularization of the iris a possibility, although neovascularization is usually a late complication. Additionally, low-dose irradiation induces an increased expression of angiostimulatory growth factors including VEGF both in the tumor microenvironment and systemically [24, 25]. Upregulation of these factors could stimulate neovascularization in a shorter time frame. The patient’s elevated intraocular pressures are most likely secondary to a history of ocular hypertension, although the inflammation from the uveitis may be a contributing factor.

### Polypharmacy in relation to ocular symptoms

In the second and third cases, polypharmacy may play a role in the manifestation of ocular symptoms as both patients had mostly unremarkable or stable eye exams despite the new onset of symptoms. The third case includes a patient on several medications during the time of Ra-223 administration including enzalutamide. Single case reports of posterior reversible encephalopathy syndrome (PRES) have been observed in individuals receiving enzalutamide [26]. PRES is characterized by seizure, headache, impaired vision, and hypertension. A relationship between PRES and cancer medications including cytotoxic chemotherapeutics and newer agents that target VEGF such as bevacizumab, sunitinib, and pazopanib has been observed [27, 28]. Case reports suggest visual disturbance symptoms 10 week to 4 months after systemic therapy initiation with these agents. In this patient’s case enzalutamide was started 14 days prior to Ra-223, with onset of ocular symptoms within 48 hours after Ra-223 administration.

In the case of the second patient, the patient is concurrently taking two potentially problematic medications. Mirabegron, an anticholinergic, is known to cause blurry vision secondary to blockage of cholinergic stimulation to the ciliary muscle of the crystalline lens. Additionally, transient visual loss secondary to polypharmacy can occur from hypoperfusion or fluctuations in ocular blood flow. The patient is taking tadalafil, which may have predisposed this patient to develop symptoms. This patient had been on these medications for several months prior to onset of blurry vision.

In both of these cases, the addition of Ra-223 with another medication may have lowered the threshold for ocular complications to occur in an additive or synergistic manner, since otherwise ocular examination in both cases were unremarkable. Should a correlation exist, it is imperative to recognize the possibility and consequence of polypharmacy, and perhaps these patients should be temporarily suspended from receiving other non-pertinent medications around the time of administration of Ra-223 or receive appropriate risk counseling.

### Poor underlying ocular health

Of the patients presented, 2 of 3 had poor underlying ocular health which may have predisposed the patient to ocular complications. The first patient had a history of bilateral cataracts and ocular hypertension; the second patient’s history was notable for monocular strabismic amblyopia, bilateral macular drusen and bilateral narrow angles. There is a known tendency for ocular comorbidities (e.g., cataracts, glaucoma, macular degeneration) to be associated with development of other ocular conditions and manifestations [29]. One case–control study found that patients with a previous diagnosis of open angle glaucoma were more likely to develop neovascular age-related macular degeneration [30]. It has also been shown that ocular manifestations such as posterior capsular tear, hyphema, macular edema, and retinal detachment following ophthalmic procedures are more common in patients with underlying comorbidities [31]. Therefore, ocular manifestations with the use of Ra-223 are conceivably more likely in patients with underlying ocular comorbid conditions.

### Biologic considerations

Ra-223 is known to preferentially incorporate into newly formed bone matrix, including within osteoblastic metastatic lesions. High energy alpha particles emitted induce DNA double-strand breaks, leading to cell death in tumor cells and nearby osteoblasts and osteoclasts. Disruption of these cells and signaling pathways, alters feedback loops of morphogenetic proteins such as TGFB, insulin-like growth factor 1, platelet-derived growth factor, endothelin-1 and VEGF. An intimate connection between angiogenesis and osteogenesis is known to exist [32, 33]. As is well established, VEGF has been shown to be an endothelial cell-specific mitogen and angiogenic inducer [34, 35]. Antibodies that bind to all subtypes of VEGF, including bevacizumab, are used to reduce macular edema, vascular permeability, retinal neovascularization, and ultimately restore vision [36]. The authors postulate that perhaps the incidence of blurry vision in relation to Ra-223, may be a result of the impact of radiation on the production of VEGF, manifesting as ocular toxicities including in the form of hyphema.

As aforementioned, in canines, the use of Ra-223 caused ocular toxicities, thought to be secondary to the calcium-receptors in the retinal epithelial layer, the tapetum lucidum [12]. While humans do not contain this reflective layer, the human retinal pigment epithelium *is* coupled with the activity of calcium channels. Excessive stimulation or interference in this epithelial monolayer can disrupt vision processes [37]. Although the dose of Ra-223 that precipitated ocular toxicities in animal studies was 3–9 times what is used clinically in humans, perhaps certain individuals may be sensitive to this interference.

Finally, a recent study by Mao et al. looked to characterize oxidative damage and apoptosis in retinal endothelial cells after exposure to gamma, proton and oxygen (^16^O) radiation to better understand why individuals may experience degradation of vision as a result of space travel. After exposure to radiation, mice eyes were isolated and examined for endothelial nitric oxide synthase (eNOS) expression and apoptosis within the retina 2 weeks after radiation therapy. Radiation exposure with low dose ^16^O, as little as 0.1 Gy exposure, elicited increased apoptosis within retinal endothelial cells. This data suggests significant changes may occur in retinal endothelial cells with low dose ionizing radiation therapy [38]. The maximum distance traveled of alpha particles is about 100 μm, so that the skeletal endosteum receives a dose coefficient of about 16 Gy while bone marrow receives 1.5 Gy. Therefore, it is feasible that the retina may receive a small dose of ionizing radiation therapy leading to ocular deficits [6].

## Conclusion

As the usage of Ra-223 potentially expands to use in other high-volume osteoblastic metastatic disease such as in hormone-receptor positive breast cancer, osseous tumors, multiple myeloma, differentiated thyroid cancer and renal cell carcinoma patients, it is imperative the full side effect profile is recognized and understood to potentially avoid adverse events [39–41]. In this case series, ocular consequences are experienced in three patients. The authors postulate that these clinical changes may be a result of radiation’s potential effect on neovascularization, poor ocular health, polypharmacy, and/or the biomolecular effects of radium-223. We present the first cases of potential ocular toxicities in those receiving Ra-223 for the first time in hopes to shed light on possible clinical adverse events in relation to this therapeutic treatment option.

## Data Availability

Not applicable.

## Abbreviations

AE: Adverse event
ADT: Androgen deprivation therapy
ERM: Epiretinal membranes
EBRT: External beam radiation therapy
HTN: Hypertension
mCRPC: Metastatic castration-resistant prostate cancer
OD: Oculus dextrus
OS: Oculus sinister
PRES: Posterior reversible encephalopathy syndrome
PCa: Prostate cancer
PSA: Prostate-specific antigen
Ra-223: Radium-223
RALP: Robot-assisted laparoscopic prostatectomy
TGF-B: Transforming growth factor B
VEGF: Vascular endothelial growth factor
